# A preliminary survey of feline filarial parasites in Kerala State, India

**DOI:** 10.1007/s00436-026-08639-9

**Published:** 2026-03-16

**Authors:** P. Preena, Mevin Sabu Mathews, V. P. Hana, Sachin Manoj, Athira Sajeendran, Anita K. Santhosh, Amrutha Anand, Adithya Sasi, K. S. Athira, Y. Ajith, Bindu Lakshmanan, S Ajithkumar, P. V. Tresamol

**Affiliations:** 1 Department of Teaching Veterinary Clinical Complex, College of Veterinary and Animal Sciences (CVAS), Kerala Veterinary and Animal Sciences University (KVASU), Kerala Mannuthy, India; 2 Department of Veterinary Clinical Medicine, Ethics and Jurisprudence, CVAS, KVASU, Mannuthy, Kerala India; 3 Department of Veterinary Parasitology, CVAS, KVASU, Mannuthy, Kerala 680 651 India; 4 Department of Veterinary Epidemiology and Preventive Medicine, College of Veterinary and Animal Sciences, KVASU, Mannuthy, Kerala India

**Keywords:** *Brugia malayi*, Cat, *Dirofilaria repens*, 5.8S–ITS2–28S ribosomal region, Microfilaria, Phylogeny

## Abstract

Feline filarial parasites remain among the least investigated in India, despite the well-established endemicity of filariosis in canine and human populations. This study aimed to assess filarial infections in domestic cats in Kerala using both microscopy and molecular techniques, with a focus on evaluating their potential role as reservoir hosts. From September 2023 to February 2024, 300 owned cats presented to the University Veterinary Hospital, Mannuthy, were screened for circulating microfilariae using wet blood film examination. Four cats (1.33%) tested positive, of which three harbored sheathed microfilariae and one unsheathed. PCR amplification targeting the 5.8S–ITS2–28S ribosomal region of ribosomal DNA identified the sheathed forms as *Brugia malayi* and the unsheathed form as *Dirofilaria repens*. No co-infections were detected. Phylogenetic analysis of the 5.8S–ITS2–28S ribosomal sequences revealed that the *B. malayi* isolates clustered with previously reported feline isolates from Thailand, suggesting regional genetic relatedness. The *D. repens* isolate showed high sequence similarity to other Indian isolates, indicating genetic continuity across local strains. Although the parasitemia was lower than that reported in dogs and in cats from other endemic countries, the detection of zoonotic species in asymptomatic cats supports further investigation of the epidemiological significance of feline infections. Comprehensive, long-term studies involving molecular surveillance and vector ecology are recommended to clarify the epidemiological significance of feline hosts in filarial parasite transmission.

## Introduction

Filariosis is a vector-borne disease caused by nematodes of the family Onchocercidae, with worldwide distribution. The filarids that parasitize animals with zoonotic potential belong to the genera *Dirofilaria*,* Onchocerca*,* Brugia*,* Dipetalonema*, and *Acanthocheilonema* (Moraes et al. 2022). Filariosis can be broadly classified based on the anatomical predilection: lymphatic filariosis, caused by *Wuchereria bancrofti*,* Brugia malayi*,* B. pahangi*, and *B. timori;* subcutaneous filariosis, caused by *Dirofilaria repens*,* Onchocerca volvulus*,* Loa loa*,* Mansonella streptocerca*, *Cercopithifilaria grassi*, and *Acanthocheilonema reconditum;* and cardiopulmonary filariosis, caused by *D. immitis* (Laidoudi et al. 2020; Mathison et al. 2019). Lymphatic filariosis is recognised as a neglected tropical disease, affecting 51.4 million people worldwide (WHO 2021). The WHO roadmap for 2030 emphasizes lymphatic filariosis elimination strategies based on mass drug administration (MDA), surveillance, and vector control interventions (WHO 2020). Filarial nematodes are transmitted by various blood-sucking arthropods, including mosquitoes, biting midges, blackflies, deer flies, and, in some cases, ticks. Within the vector, the ingested L1 microfilarial stage develops into the infective L3 larva, which is then transmitted to a new host during subsequent blood meals (Kaikuntod et al. 2020).

Among companion animals, canine filariosis is well documented across various parts of India, with notably high prevalence rates. In Kerala, infection rates in dogs have reached 80% in Thrissur and 42.68% in Alappuzha districts (Ravindran et al. 2014). Similarly, Abd Rani et al. (2010) reported widespread canine filarial infections in other regions of the country. In contrast, feline filariosis remains understudied, though reports from several Asian countries indicate its presence. Studies from Malaysia, Thailand, and Sri Lanka suggest that the prevalence of *Brugia* spp. is consistently higher in dogs than in cats (Naing et al. 2024). In Sri Lanka, Mallawarachchi et al. (2018) reported high filarial infection rates in both dogs (68.8%) and cats (47.8%). In Malaysia, *B. pahangi* and *D. repens* were identified as the predominant filarial species in cats, with an overall prevalence of 23.5%. Among infected cats, 35% were positive for *B. pahangi*, 50% for *D. repens*, and 15% had co-infections (Al-Abd et al. 2015).

Despite well-documented reports of filariosis in dogs, data on feline filarial infections in India remain scarce. To our knowledge, no large-scale studies have assessed the prevalence or diversity of filarial parasites in domestic cats in the country. Meanwhile, increasing numbers of feline filarial infections have been documented globally, including in Southeast Asia (Baticados et al. 2013). The potential of cats to serve as reservoirs for zoonotic filarial parasites remains underexplored, particularly in endemic regions like India. A better understanding of feline filariosis could contribute to improved epidemiological knowledge, aid in risk assessment, and support the development of effective control strategies for both animal and human health. Such data are vital for veterinarians and public health professionals in designing surveillance and intervention programs (Al-Abd et al. 2015). Therefore, the present study aimed to investigate the occurrence and molecular characterization of filarial parasites in domestic cats in Kerala, India, using parasitological screening and PCR-based identification.

## Materials and methods

### Sample collection

A six-month survey was conducted from September 2023 to February 2024. Domestic cats of various ages and breeds presented to the University Veterinary Hospital, Mannuthy (Thrissur, Kerala), for routine procedures such as check-ups, vaccinations, wound dressing, or treatment of minor ailments were included in the study. Cats were physically restrained by their owners, or, in difficult cases, placed in cages or cat bags. The tip of the ear was shaved and disinfected with medicated spirit. Capillary blood was collected by pricking the ear margin using a sterile 24-gauge needle.

### Detection of circulating microfilaria

All animals were screened for microfilariae using wet blood film examination of peripheral capillary blood. A drop of blood from each cat was placed onto a clean glass slide and immediately examined under low-power (4×) light microscopy for motile microfilariae. Thick blood smears were prepared from positive samples, stained using Field’s stain (Nice Chemicals, India), and submitted to the Department of Veterinary Parasitology, CVAS, KVASU, Mannuthy, for species-level identification based on morphological features and the presence or absence of a sheath (Mallawarachchi et al. 2018).

### Molecular analysis

Venous blood (0.5 mL) was aseptically collected from microfilaremic cats into EDTA vials via femoral venipuncture following appropriate restraint. Genomic DNA was extracted from 100 µL of EDTA-buffered whole blood using the DNeasy^®^ Blood and Tissue Kit (Qiagen, Germany) according to the manufacturer’s instructions. Molecular screening for filarial parasites was performed using panfilarial primers DIDR-F1 (5′-AGT GCG AAT TGC AGA CGC ATT GAG-3′) and DIDR-R1 (5′-AGC GGG TAA TCA CGA CTG AGT TGA-3′), which amplify a segment of the 5.8S–ITS2–28S ribosomal region, allowing differentiation of *D. immitis*,* D. repens*,* B. malayi*,* B. pahangi*,* A. reconditum*,* and A. dracunculoides* (Rishniw et al. 2006). Positive control DNA for *D. repens* and *B. malayi* (obtained from the Department of Veterinary Parasitology, CVAS, KVASU, Mannuthy) and sterile nuclease-free water (negative control) were included in each PCR run. PCR was performed in a 20 µL reaction volume containing 10 µL of EmeraldAmp^®^ GT PCR Master Mix (2X) (TaKaRa Bio, Japan), 1 µL of DNA (50 ng/µL), and 1 µL of each primer (10 pM). Amplification was carried out using a thermal cycler (T100™, Bio-Rad) with the following conditions: initial denaturation at 95 °C for 5 min; 35 cycles of denaturation (94 °C for 30 s), annealing (59 °C for 30 s), and extension (72 °C for 30 s); followed by a final extension at 72 °C for 10 min. PCR products were electrophoresed on a 2% agarose gel in 1× TAE buffer at 70 V and visualized using a gel documentation system (Bio-Rad Laboratories, USA). Species identification was based on amplicon size (Rishniw et al. 2006). PCR-positive products were sequenced using Sanger’s method at GeneSpec Biosciences, Kochi.

### Sequencing and phylogenetic analysis

The obtained sequences were analyzed using the nBLAST tool on the NCBI database to determine similarity with previously reported gene sequences. For phylogenetic analysis, 5.8S–ITS2–28S ribosomal region sequences of *Brugia* and *Dirofilaria* spp. were compared with corresponding sequences available in GenBank. The dataset included isolates from a wide range of hosts and geographical regions to ensure comprehensive comparison. *Brugia* spp. sequences were obtained from dogs, cats, and humans in countries such as Thailand, the USA, Indonesia, and India. *Dirofilaria* spp. sequences were retrieved from dogs, cats, and red panda samples originating from India, China, Iran, and the USA. Relevant sequences of other filarial species like *O. fasciata* (camel, China), *S. digitata* (cattle, Sri Lanka), and *A. reconditum* (dog, Taiwan) were included as outgroups. Representative nucleotide sequences obtained in the present study were submitted to the GenBank database.

The evolutionary history was inferred using the Maximum Likelihood method with the Tamura 3-parameter model (Tamura 1992) in MEGA X (Kumar et al. 2018). Initial trees for the heuristic search were generated automatically by applying the Neighbor-Joining and BioNJ algorithms to a matrix of pairwise distances estimated using the same model, and selecting the topology with the highest log-likelihood.

### Data analysis

Data were entered into a Microsoft Excel spreadsheet and exported into SPSS version 24.0 for Windows (IBM Corp., Armonk, NY) for analysis. Frequencies and proportions were calculated for descriptive statistics.

## Results

### Prevalence and identification of microfilariae in domestic cats

**Fig. 1:**
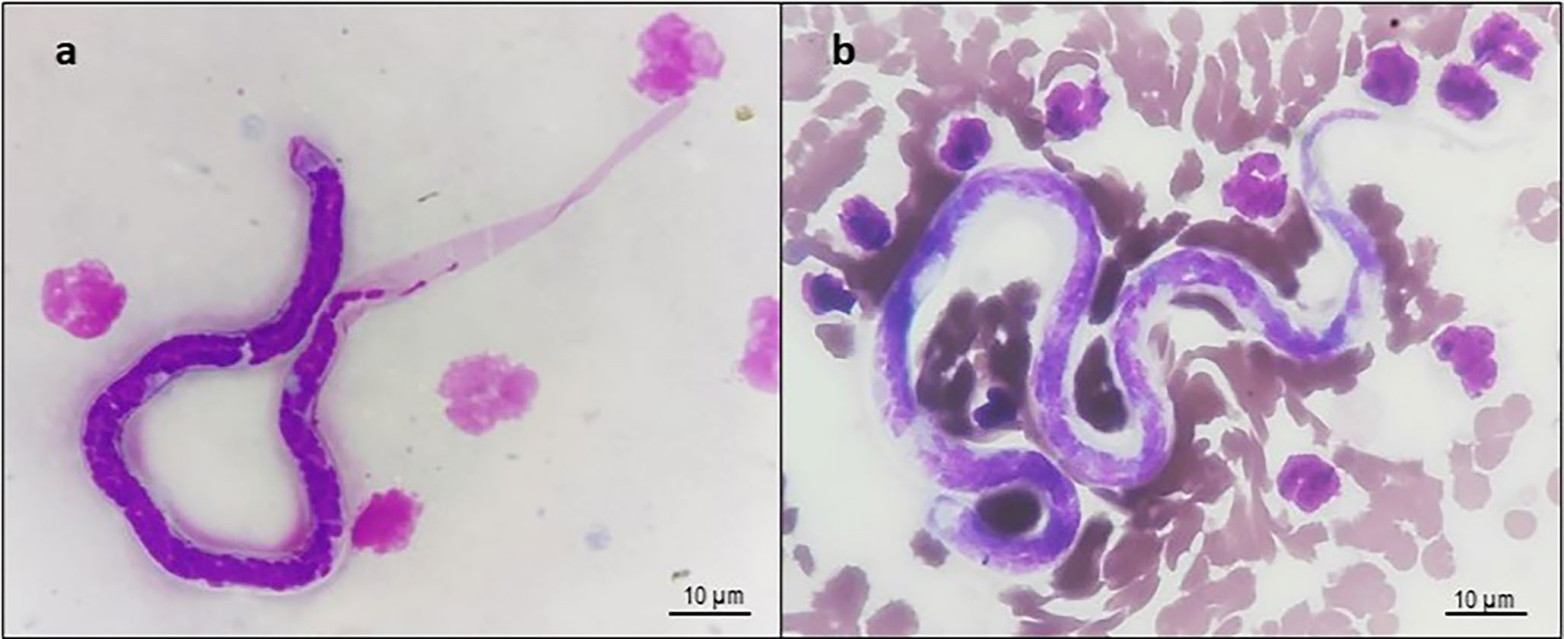
**a** Sheathed microfilaria of *Brugia* spp. surrounded by leucocytes in the background; **b** Unsheathed microfilaria of *D. repens* amidst red blood cells and leucocytes in the background (Field’s stain; 1000x magnification). The scale bar in both panels represents 10 μm.

A total of 300 domestic cats were screened, comprising 157 Persian and 143 non-descript breeds, with a male-to-female ratio of 1:1. The age distribution was as follows: 129 cats were aged < 1 year, 133 were 1–3 years old, and 38 were > 3 years. Of the 300 cats examined using peripheral blood wet film examination, four cats (1.33%; 95% CI: 0.04-2.63%) tested positive for circulating microfilariae. Among them, three cats (0.99%) had sheathed microfilariae, identified morphologically as *Brugia* spp., and one cat (0.33%) had unsheathed microfilariae, identified as *D. repens* (Fig. 1a and b). All four microfilaremic cats were male. Three were between 1 and 3 years old, and one was younger than 1 year. These cats were presented for unrelated clinical conditions, such as vomiting, trauma, or respiratory distress. None exhibited clinical signs attributable to filariosis.

**Fig. 2:**
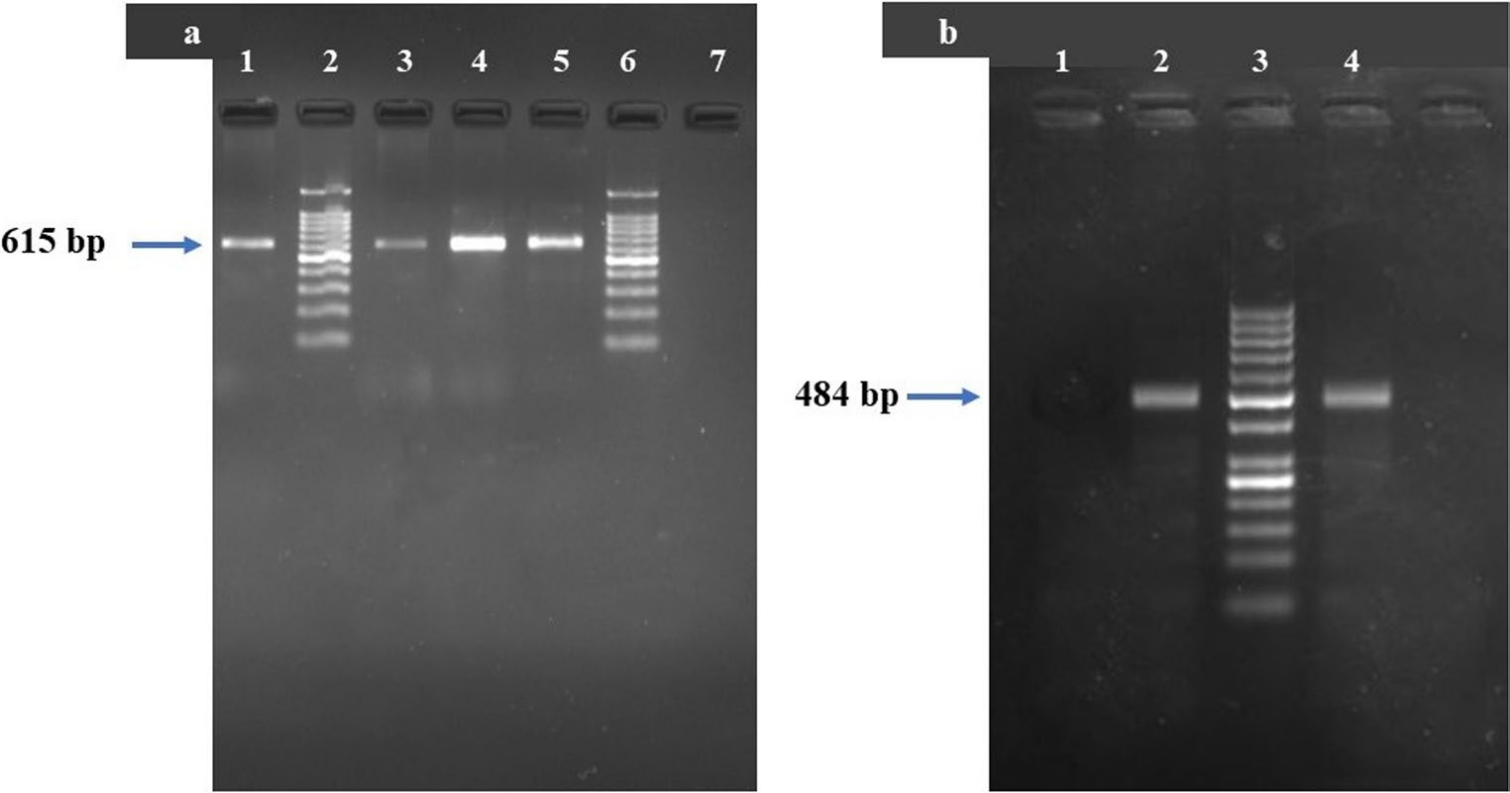
Gel electrophoresis of PCR products following amplification with panfilarial primers. **a** Lane 1: *B. malayi* positive control; Lane 2 & 6: 100 bp ladder; Lanes 3, 4 & 5: Positive *B. malayi* cat DNA samples , Lane 7: Negative control; **b** Lane 1: Negative control, Lane 2: *D. repens* positive control, Lane 3: 50 bp ladder; Lane 4: Positive *D. repens* cat DNA sample.

PCR analysis using panfilarial primers confirmed that the sheathed microfilariae belonged to *B. malayi* , indicated by amplification at 615 bp, while the unsheathed microfilaria was identified as *D. repens*, with a band at 484 bp (Fig. 2a and b). No co-infections with either species were detected in any of the samples.

### Sequence analysis and phylogenetic relationship

Three sequences obtained in this study namely, A_F14677, B_F16446, and C_F614 were submitted to the GenBank database under the accession numbers PQ032437, PQ032438, and PQ032439, respectively. The two *B. malayi* isolates yielded identical nucleotide sequences and clustered into a single haplotype, showing 96.45% to 98.7% identity with *B. malayi* and *B. pahangi* from cats in Narathiwat province, Southern Thailand. The *D. repens* sequence showed 99.6% and 98.2% similarity with *D. repens* isolates from another district (Wayanad) of Kerala, India.

**Fig. 3:**
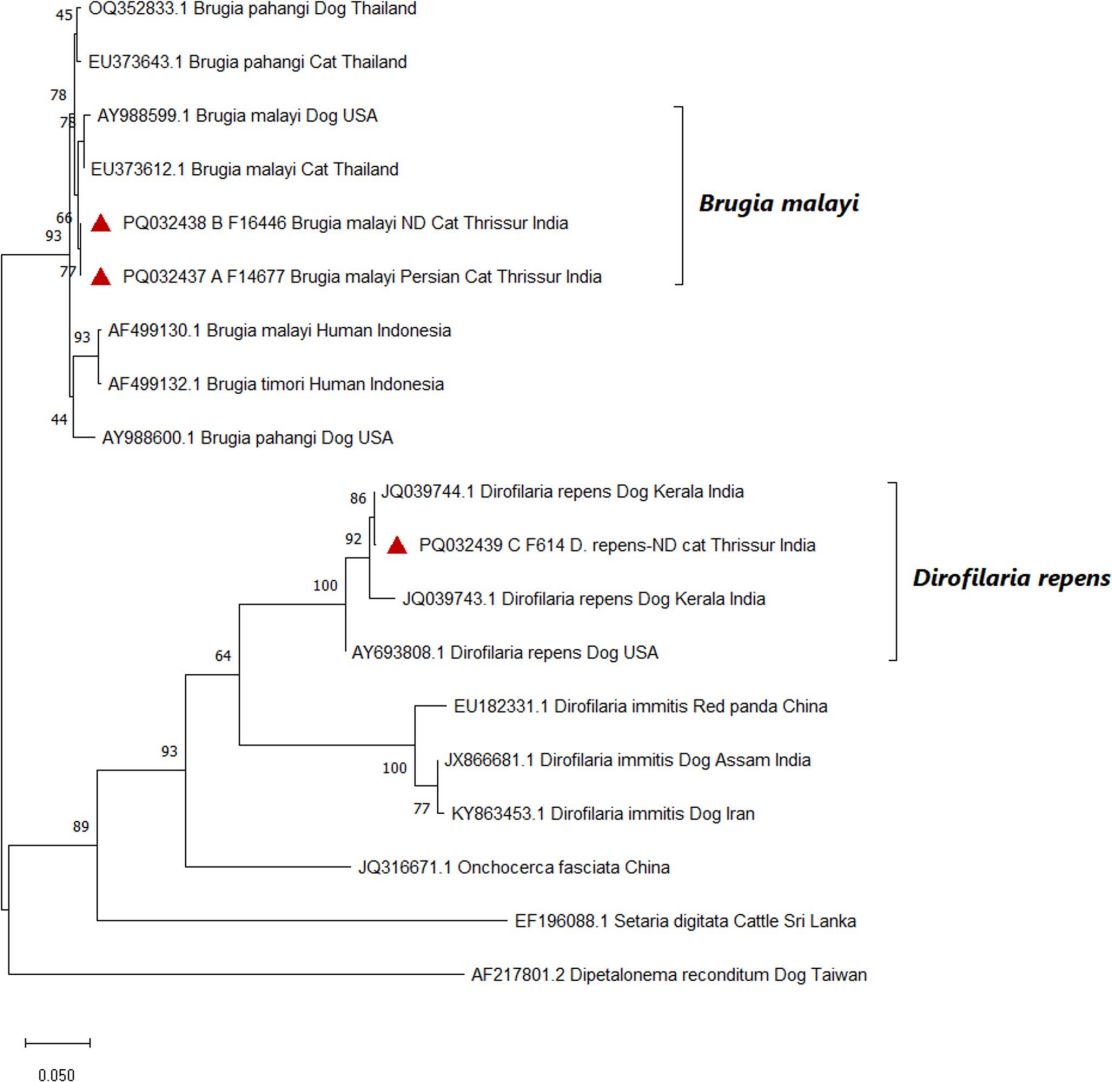
Phylogenetic analysis of *B. malayi* and *D. repens* 5.8S–ITS2–28S ribosomal region. A phylogenetic tree based on the 5.8S–ITS2–28S ribosomal region of both species in the study was inferred by using the Maximum Likelihood method and Tamura 3-parameter model. The tree with the highest log likelihood (-3705.96) is shown. Bootstrap support values are indicated at the nodes, representing the percentage of trees in which the associated taxa clustered together. The tree is drawn to scale, with branch lengths measured in the number of substitutions per site. This analysis involved 19 nucleotide sequences. There was a total of 889 positions in the final dataset. Each node is labelled with accession number, species, host and place. Sequences marked with the red triangles represent isolates obtained in the present study.

The phylogenetic relationships of the 5.8S–ITS2–28S rDNA sequences analyzed in this study are illustrated in Fig. 3. The *B. malayi* sequences A_F14677 (PQ032437) and B_F16446 (PQ032438) clustered closely, indicating minimal genetic variation and suggesting a shared lineage between *B. malayi* isolates from Persian and non-descript (ND) cats in Thrissur, India. The phylogeny placed *B. malayi* (PQ032437, PQ032438, AY988599.1, EU373612.1), *B. pahangi* (OQ352833.1, EU373643.1, AY988600.1), and *B. timori* (AF499132.1) into distinct clades, reflecting divergence across host types (cats, dogs, humans) and geographic regions (Thailand, USA, Indonesia). These findings suggest that *Brugia* spp. exhibit considerable genetic diversity associated with host specificity and geographic distribution. Similarly, *D. repens* (PQ032439, JQ039744.1, JQ039743.1, AY693808.1) and *D. immitis* (EU182331.1, KY863453.1) formed well-supported subclades, with high bootstrap values (e.g., 100, 86) indicating strong evolutionary separation among strains from India, China, and the USA. The *D. repens* sequence C_F614 (PQ032439) showed closest similarity to Indian isolates JQ039743.1 and JQ039744.1 (both from Kerala), suggesting regional genetic conservation potentially influenced by local transmission dynamics or environmental factors. The distinct clustering of C_F614 from *B. malayi* sequences emphasizes the evolutionary divergence between *Dirofilaria* and *Brugia* genera. Outgroup sequences including *O. fasciata* (JQ316671.1), *S. digitata* (EF196088.1), and *A. reconditum* (syn. *D. reconditum;* AF217801.2) were placed at the base of the tree, confirming their evolutionary distance from the core filarial genera analyzed.

## Discussion

Although companion animals are considered potential reservoirs for zoonotic filarial nematodes, there are limited data specifically investigating filarial infections in domestic cats in India. Microscopic examination of stained blood smears remains a commonly used screening tool for detecting microfilariae; however, in feline hosts, its sensitivity is limited due to typically low or transient parasitemia, and the method requires substantial expertise for species differentiation. Moreover, microscopy cannot reliably distinguish closely related filarial species (Roblejo-Arias et al. 2023). In this study, wet film microscopy was used as an initial screening method due to its practicality in a clinical setting. However, its limitations in cats are acknowledged. More sensitive techniques, such as the modified Knott’s test or PCR, are better suited for accurate detection, especially in cases with low microfilaremia. Consequently, the prevalence reported here likely represents an underestimate of the true infection rate, owing to the inherent low sensitivity of wet film microscopy for detecting feline microfilaremia. We report here the first molecular confirmation of *B. malayi* and *D. repens* in domestic cats from India, supported by both morphological and sequence-based evidence.

The study period, from September 2023 to February 2024, was conventionally chosen and not designed to account for potential seasonal variation in vector populations. Previous studies have shown that *D. repens* is endemic in dogs in Kerala, and co-infections with *Brugia* species have also been reported (Ravindran et al. 2014; Edana et al. 2023). It is possible that infected dogs in shared environments may serve as a source of microfilariae for domestic cats, warranting further epidemiological investigation. Given the small number of positive cases and the hospital-based sampling approach, the generalizability of the findings is limited, and the results may not accurately reflect the true prevalence of feline filariosis in the broader cat population. Wet film microscopy, used as the primary screening method, has low sensitivity particularly in cats compared to modified Knott’s test or PCR, and likely led to underestimation of prevalence.

While preliminary species differentiation was performed microscopically based on sheath presence, microscopy alone cannot reliably confirm species identity, as the method is labor-intensive, requires considerable expertise, and is prone to misidentification of closely related species. PCR with panfilarial primers followed by sequencing provided more accurate confirmation. Cats are known to be imperfect hosts for filarial infections: they often develop occult infections due to immune-mediated clearance of microfilariae or reversible suppression of microfilarial production, resulting in transient or absent microfilaremia. They also exhibit biological features that complicate diagnosis, including low parasite burdens, single-sex (male) infections, and a short adult worm lifespan (American Heartworm Society 2014). Antigen detection tests are often ineffective in identifying occult infections, while antibody tests indicate exposure but do not confirm active infection (Hoch and Strickland 2008). Additionally, amicrofilaremia has been reported in hosts where larvae fail to develop into mature worms, potentially explaining undetected infections (Edeson et al. 1960).

Similar to the present study, Ravindran et al. (2014) reported a higher prevalence of filarial infections in male dogs and those older than two years, suggesting possible sex- or age-related risk factors. In the southeastern United States, Atkins et al. (2005) found that non-domestic cats were at risk for heartworm infection, with male cats showing higher exposure rates, further supporting the observed male bias. In Thailand, Nuchprayoon et al. (2006) identified filarial parasites in 5 of 52 cats (9.5%), including *B. pahangi* (7.6%) and *D. immitis* (1.9%), confirming the presence of both zoonotic filariae in felines. By contrast, a study in Makati City, Philippines, by Baticados et al. (2013), found that none of the cats exhibiting clinical signs consistent with dirofilariosis such as coughing, dyspnea, vomiting, diarrhea, anorexia, and weight loss tested positive for circulating microfilariae or heartworm antigens. This apparent disconnect may be attributed to limitations of antigen tests, which are highly specific for detecting antigens produced by mature female *D. immitis* worms, typically at least 7–8 months of age. These assays often fail to detect early infections (< 5 months post-infection) or male-only infections, potentially explaining false-negative results despite clinical suspicion.

Co-infections with multiple filarial species were not detected in the present study, in contrast to Mallawarachchi et al. (2018), who reported co-infection with *B. malayi* and *D. repens* in 36 dogs and 17 cats via PCR analysis. The overall prevalence of feline filariosis in this study was 1.33%, which is low compared to canine dirofilariosis, and aligns with findings by Montoya-Alonso et al. (2017). This may be partly attributed to the semi-domestic lifestyle of cats, which typically spend more time indoors than dogs (Naing et al. 2024). However, most feline filariosis studies have been concentrated in Southeast Asia, where cats have been considered potential reservoirs for zoonotic lymphatic filariosis (Mallawarachchi et al. 2018). Molecular studies support this zoonotic role. For example, Tan et al. (2011) reported identical *cox1* gene sequences of *B. pahangi* in an infected cat, a human patient, and *Armigeres subalbatus* mosquito larvae in suburban Kuala Lumpur, suggesting that domestic cats may serve as a source of zoonotic infection in endemic areas. The clinical impact of filarial infection was not assessed in our surveyed cats. However, past reports have documented clinical signs in *D. repens* infected cats, including pruritus, cutaneous lesions, and concurrent hemobartonellosis (Tarello 2002). In lymphatic filariosis, cats have shown symptoms resembling human cases like limb edema and lymphadenopathy (Mallawarachchi et al. 2018). Subcutaneous filariosis due to *D. repens* may manifest as nodules, papular or crusting dermatitis, eosinophilia, and eosinophilic lymphadenitis, especially in the axillary and inguinal lymph nodes (Pennisi et al. 2020). As noted in the present study, the infected cats were presented with unrelated clinical issues, and none exhibited signs typically associated with feline filariosis, which is consistent with the subclinical nature of *B. malayi* and *D. repens* infections in cats. In cases of cardiopulmonary filariosis, clinical signs are often absent, but when present, may include coughing, dyspnoea, vomiting, diarrhoea, lethargy, and weight loss. Severe cases may involve neurological signs or sudden death, although heart murmurs are rare due to the uncommon development of right-sided cardiomegaly and caval syndrome in cats (Hoch and Strickland 2008).

Therapeutically, Sarasombath et al. (2019) recommended topical selamectin at 6 mg/kg every two months for treating *B. malayi* infections, and twice-yearly applications for chemoprophylaxis. Taweethavonsawat and Chungpivat (2013) reported successful treatment of *B. pahangi* with weekly ivermectin injections (400 µg/kg) for two months. In canine models, Bazzocchi et al. (2008) demonstrated that a combination of ivermectin and doxycycline is effective against *D. immitis*, with ivermectin targeting both microfilariae and adult worms, and doxycycline eliminating *Wolbachia*, the symbiotic bacterium essential for worm survival and reproduction.

The phylogenetic analysis provides insights into the genetic diversity and evolutionary relationships within *Brugia* and *Dirofilaria* species. The high bootstrap values supporting species-specific clades indicate strong genetic similarity across isolates from different regions and hosts. This suggests evolutionary stability in the ITS2 gene region, supporting its use as a reliable marker for phylogenetic analysis of filarial nematodes. The clustering of *B. malayi* and *B. pahangi* sequences from diverse hosts suggests minimal genetic divergence, potentially reflecting cross-host transmission dynamics or limited host-specific adaptation. Likewise, the conserved clustering of *D. repens* sequences from geographically distant regions indicates a stable genetic structure, despite environmental variation. These findings are consistent with previous reports highlighting the evolutionary conservation of the ITS2 region among filarial species. For instance, Abd Rani et al. (2010) reported both morphological and molecular evidence supporting ITS2-based phylogenetic placement of a novel *Acanthocheilonema* species in dogs from Ladakh. Conversely, Areekit et al. (2009) noted that intraspecies variation in ITS2 sequences may differ significantly across filarial genera, making direct comparisons between species more complex. Overall, these results emphasize the need for further investigation into the ecological and epidemiological factors influencing the observed genetic stability of filarial nematodes across different hosts and geographic regions.

This study has several limitations that warrant consideration. The number of positive cases was low, limiting broader conclusions about the true prevalence of feline filariosis in the region. Sample collection was confined to cats presented to a veterinary teaching hospital, which may not represent the wider feline population and introduces potential selection bias. Microscopy was used as the primary screening tool; although preliminary species differentiation was attempted based on sheath presence, this method has low sensitivity in cats due to typically transient or absent microfilaremia and cannot reliably distinguish closely related species without considerable expertise. Although PCR was used for molecular confirmation, it was applied only to microscopy-positive samples, which may have further underestimated prevalence. With respect to phylogenetic analysis, a few GenBank entries labelled as *Dirofilaria sp. hongkongensis* (now considered synonymous with *D. asiatica*) were identified for potential comparison; however, these sequences are short partial 5.8S–ITS2-28S fragments (e.g., PQ728425.1, PQ728424.1, PQ728423.1 and PQ728422.1) of only ~ 300 bp and do not sufficiently overlap with the region amplified in our study. As a result, they could not be incorporated into the ITS2-based phylogenetic reconstruction, limiting the ability to visually assess separation between *D. repens* and the Hong Kong/asiatica genotype. Nevertheless, the identity of our isolate as *D. repens* is strongly supported by two key molecular findings: the consistent amplification of the species-specific 484 bp 5.8S–ITS2–28S amplicon, and the ≥ 98–100 sequence identity established through comparison with established Indian *D. repens* isolates, providing robust evidence against misidentification. Finally, the absence of entomological surveillance limited our ability to identify local vectors, which is essential for understanding regional transmission dynamics. Community-based sampling with molecular screening is warranted to refine prevalence estimates and clarify the epidemiologic role of feline infections.

## Conclusion

This study reports a low prevalence of two filarial nematodes, *B. malayi* and *D. repens,* in domestic cats from Kerala, India, based on combined morphological and molecular evidence. While all infected cats were clinically asymptomatic, the detection of th-ese zoonotic species in felines highlights the need for further epidemiological studies to better understand their potential role in transmission dynamics. Although the role of cats as reservoirs remains uncertain, their involvement in the local parasite ecology cannot be entirely excluded. Future research should explore vector populations, particularly mosquitoes, and assess co-infections in other hosts such as dogs to build a clearer picture of transmission cycles. Current public health and zoonoses control strategies may benefit from considering the possible, though likely limited, involvement of domestic cats in endemic regions.

## Data Availability

All data are presented in the manuscript.
